# Phytalgic^®^, a food supplement, vs placebo in patients with osteoarthritis of the knee or hip: a randomised double-blind placebo-controlled clinical trial

**DOI:** 10.1186/ar2891

**Published:** 2009-12-16

**Authors:** Alain Jacquet, Pierre-Olivier Girodet, Antoine Pariente, Karelle Forest, Laurent Mallet, Nicholas Moore

**Affiliations:** 1 Department of Pharmacology, University of Bordeaux, 146 rue Léo Saignat, 33076, Bordeaux, France; 2 CHU de Bordeaux, 12 rue Dabernat, 33404 Talence, France; 3 Centre d'Investigation Clinique, INSERM CIC0005, 146 rue Léo Saignat, 33076, Bordeaux, France; 4 Phythea, 13 Rue Elsa Triolet, 77176 Savigny, France

## Abstract

**Introduction:**

The medicinal treatment of osteoarthritis (OA) is mostly symptomatic to relieve pain and incapacity with analgesics and non-steroidal anti-inflammatory drugs (NSAIDs), drugs with well-known risks. Complementary medicines might reduce the symptoms of OA and decrease the need for NSAIDs. This study tested the effects of a food supplement, Phytalgic^®^, on pain and function in patients with osteoarthritis and their use of analgesic and NSAIDs.

**Methods:**

A randomized double-blind parallel-groups clinical trial compared Phytalgic^® ^(fish-oil, vitamin E, *Urtica dioica*) to a placebo for three months, in 81 patients with OA of the knee or hip using NSAIDs and/or analgesics regularly. The main outcome measures were use of NSAIDs (in Defined Daily Doses per day - DDD/day) or analgesics (in 500 mg paracetamol-equivalent tablets per week (PET/week) measured each month, and Western Ontario-McMaster University Osteo-Arthritis Index (WOMAC) function scales.

**Results:**

After three months of treatment, the mean use of analgesics in the active arm (6.5 PET/week) vs. the placebo arm (16.5) was significantly different (*P *< 0.001) with a group mean difference of -10.0 (95% CI: -4.9 to -15.1). That of NSAIDs in the active arm (0.4 DDD/day) vs the placebo arm (1.0 DDD/day) was significantly different (*P *= 0.02) with a group mean difference of - 0.7 DDD/day (95% CI: -0.2 to -1.2). Mean WOMAC scores for pain, stiffness and function in the active arm (respectively 86.5, 41.4 and 301.6) vs the placebo arm (resp. 235.3, 96.3 and 746.5) were significantly different (*P *< 0.001) with group mean differences respectively of -148.8 (95% CI: -97.7 to -199.9), -54.9 (95% CI: -27.9 to -81.9) and -444.8 (95% CI: -269.1 to -620.4).

**Conclusions:**

The food supplement tested appeared to decrease the need for analgesics and NSAIDs and improve the symptoms of osteoarthritis.

**Trial registration:**

Clinicaltrials.gov NCT00666523.

## Introduction

Osteoarthritis is one of the more common of the chronic degenerative diseases afflicting an increasingly older population. It affects primarily the weight-bearing joints (knee, hip), causing pain, stiffness and impotence, ultimately crippling patients and reducing quality of life. There is no curative treatment for this disease, and its final outcome is often joint replacement of the hip or knee. However, because of the limited life expectancy of these replacements, they are usually postponed as much as possible. In the meantime, symptomatic pain relief can be obtained with analgesics such as paracetamol, or the more effective non-steroidal anti-inflammatory drugs (NSAIDs) [1-3]. NSAIDs, while generally safe when used at low doses and short term [4-6], can result in serious complications (gastrointestinal bleeding, renal failure, coronary heart disease) when used long-term or at higher doses in elderly patients, which is often the case in patients with osteoarthritis [7-9]. Different products have been proposed for long-term symptomatic relief of osteoarthritis, some of them purporting to have some chondroprotective effects, such as chondroitin sulphate, diacerhein or glucosamine [10-13]. Though meta-analyses are not very encouraging for direct effects of these adjuncts [14], we found that chondroitin sulphate might be associated with less use of analgesics or NSAIDs [15]. Some food supplements might also have effects on osteoarthritis symptoms [16-21], but few of these studies are methodologically valid. Authors have recommended testing fish-oils in patients with arthritis, and supplementation with minerals such as zinc or selenium, and vitamin E [22].

Patients using one such marketed multi-component food supplement reported reduced use of NSAIDS, at least for some time. Though it seemed highly unlikely that this supplement could suppress symptoms of OA, decreasing the use of NSAIDs might be a desirable objective in these high-risk long-term users. We therefore performed a randomized double-blind placebo-controlled clinical trial of this marketed food supplement, on the use of analgesics and NSAIDs on pain, stiffness and function scales of osteoarthritis to ensure that any decrease in analgesic and anti-inflammatory use was not at the expense of patient function.

## Materials and methods

### Study design and participants

This was a double-blind, randomized, placebo-controlled, parallel-arms clinical trial.

It was approved by the Regional Bordeaux B Committee for the Protection of Patients, and was registered with clinicaltrials.gov (NCT00666523). All patients gave written informed consent before random assignment to product or placebo. This study was performed according to all national laws and regulations governing the conduct of Clinical Trials. It conformed to the revised declaration of Helsinki, and to Good Clinical Practice.

The objective of the study was to test the effects of the food supplement combination on symptoms of osteoarthritis and on the use of analgesics and NSAIDs, with the prior hypothesis that the preparation would decrease the symptoms by an unspecified amount, and reduce the use of analgesics and NSAIDs by at least 20% from initial usage.

Patients were considered eligible if they had chronic osteoarthritis of the knee or hip (as evidenced from clinical history and X-ray documentation), experienced pain and/or stiffness requiring the regular use of NSAIDs, were aged between 40 and 80 years of age, and could demonstrate their capacity to fill a diary relating their daily treatment.

Patients with inflammatory arthritis, with osteoarthritis not affecting the knees or hips, with known allergy to any of the constituents of the study drug, with a life expectancy shorter than the duration of the study, who were pregnant or lactating, or who were not legally fit to participate in such a study were not included.

Osteoarthritis (OA) was diagnosed by the referring rheumatologist according to the American College of Rheumatology (ACR) criteria for osteoarthritis of the hip or knee. OA could be degenerative or post-traumatic.

### Study procedures

Undistinguishable numbered treatment units were prepared according to a randomization list using random block sizes. Patients were included sequentially. Blinding was lifted only after database lock.

After a four-week run-in phase during which eligible patients were trained to use the diary and tested for severity of osteoarthritis and use of analgesics or NSAIDs, patients meeting all inclusion criteria were given blinded, randomized treatment by the study drug or an indistinguishable placebo, for three successive four-week periods. They returned for consultation and assessment at the end of each period. They filled a diary in which they indicated each day all medication used (study drug and other medication). A single investigator within the Department of Pharmacology, Université Victor Segalen, Bordeaux, France followed all patients.

The study was conceived as an add-on study to the usual symptomatic medication (analgesics and/or NSAIDs). Patients were asked not to modify the nature of their treatment for pain and stiffness control and to keep using their usual analgesic and NSAID medications as often as needed during the treatment period. The study drug was used in addition to these medications. Patients were told that they could reduce the use of their pain and inflammation medication if they did not feel the need for them, but they had to note all drugs taken every day in their diary.

The study treatment is a commercially prepared food supplement, Phytalgic^®^, a marketed preparation which consists of capsules containing fish oils rich in omega-3 and omega-6 fatty acids, *Urtica dioica *(the common nettle), zinc and vitamin E; or an identical placebo. It is produced according to Good Manufacturing Practices by Phythea laboratories (77176 Savigny le Temple, France).

For the study, the manufacturer provided both the active treatment and the identical placebo, which were prepared in identical boxes distinguishable only by treatment number according to the randomisation list provided by the Department of Pharmacology Statistics Unit.

This preparation was used according to the manufacturer's recommendations, that is, three capsules per day of study drug or placebo, one in the morning, two in the evening. These are odourless and tasteless capsules. The placebo was prepared by the manufacturer with identical capsules, using non-fish oils (without omega-3 or omega-6 fatty acids) with the appropriate colourings and additives to give the placebo capsules the same consistence and colour.

Each patient was given treatment for 40 days at the beginning of each study period, and was asked to return all used containers and unused capsules at the next visit.

### Study outcome measures

#### Main study outcomes

- The use of analgesic drugs and NSAIDs, as recorded in the patient diary.

Analgesics included paracetamol alone; paracetamol combined with weak opiates (for example, coproxamol or coparein); opiates alone (morphine sulphate, dextropropoxyphene, or tramadol); low-dose NSAIDs (ibuprofen 200 mg, diclofenac 12.5 mg); short-acting NSAIDs used for analgesia (flurbiprofen, tiaprofenic acid), aspirin; analgesic use was measured in number of tablets equivalent to 500 mg paracetamol or 200 mg ibuprofen used per week. The lowest marketed adult dose of the drug was considered as approximately equivalent (paracetamol 500 mg with or without opiate, ibuprofen 200 mg, ketoprofen 25 mg, diclofenac 12.5 mg, flurbiprofen 100 mg, tiaprofenic acid 100 mg, and so on).

- Use of NSAIDs was measured as the number of defined daily doses (DDD) used over each study period, which were then summed and studied as mean daily DDD used [23-25]. Drugs were summed globally. These included traditional non-selective NSAIDs at usual anti-inflammatory doses, piroxicam (DDD 20 mg), tenoxicam (DDD 20 mg), ketoprofen (DDD 150 mg), diclofenac (DDD 100 mg), naproxen (DDD 500 mg), sulindac (DDD 400 mg), ibuprofen (DDD 1200 mg); and the COX2 selective agent: celecoxib (DDD 200 mg); though they could change the dosage, patients were asked to continue using the same drugs throughout the study.

- Western Ontario-McMaster University Osteo-Arthritis Index (WOMAC): Patients' function, stiffness and pain were assessed at each visit using the WOMAC scales. These scales explore pain, stiffness and function. They were analyzed in each of the three dimensions, and globally, by summing the scores of the individual items.

#### Secondary outcomes

- Use of slow-acting symptomatic drugs used to treat osteoarthritis: diacerhein, soy and avocado unsaponifiables, chondroitin sulphate was measured as DDD per day.

- Gastro-protective drug use, all of which were proton pump inhibitors, was also measured in DDD per day.

- Tolerability: any treatment-emergent adverse events reported by the patients were recorded (adverse events not reported previously or not present during the run-in period, or worsening during the treatment period), and the overall impact was classified into five classes: 0: no adverse event noted; 1: no treatment emergent adverse events; 2: mild and short-lasting symptoms events during the first days of the first treatment period after treatment onset; 3: mild symptoms or signs reported or observed several times during the study period, or persisting throughout the study period; and 4: manifest signs or symptoms impacting daily life, or non-mild persistent symptoms.

In addition, any serious events according to regulatory definitions were recorded and reported to the relevant authorities.

### Statistical analysis

All analyses were done in intention to treat (ITT), using all available data at each time point and baseline observation carried forward (BOCF) approach for missing data at M1, M2 and M3.

Quantitative variables were described in terms of mean ± SD, median and range, qualitative variables in terms of number and %). Parametric Student t-test or Non-parametric Mann-Whitney U test were used for quantitative variable comparisons, according to distribution characteristics. Qualitative variables were compared using Chi-square or Fischer exact test according to sample size. Ninety-five percent confidence intervals were estimated by boostrapping method. WOMAC Scores were analysed using repeated-measures analysis. Analysis of the analgesic and NSAID drug utilization was done using repeated-measures analysis of covariance, using treatment group, treatment periods, their interaction and including the WOMAC score as covariate. If the overall tested showed statistically significant differences between the active and placebo arms, the successive treatment periods would be compared. Statistical significance was set at 0.05. All analyses were done with SAS version 8.1 by the statistics group of the Bordeaux University Department of Pharmacology.

#### Sample size

The hypothesis was that compared to placebo, the active composition would result in a 20% greater decrease in the use of analgesics or NSAIDs than placebo (main study outcome). With an alpha risk set at 5%, and a power of 90% to detect this difference, it was estimated that 35 patients per treatment arm should be included. To take into account possible dropouts and missing data, it was decided to include 40 patients per study arm.

## Results

Ninety-five patients were eligible for pre-inclusion. Of these 14 were excluded during the pre-inclusion period, for personal reasons (n = 9), for insufficient severity of OA (n = 3), for excess severity of OA resulting in hospitalization (n = 1), for severe depression resulting in psychiatric hospitalization during the pre-inclusion period (n = 1).

Eighty-one included patients were therefore randomized to active treatment (41 subjects) or placebo (40 subjects).

**Figure 1:**
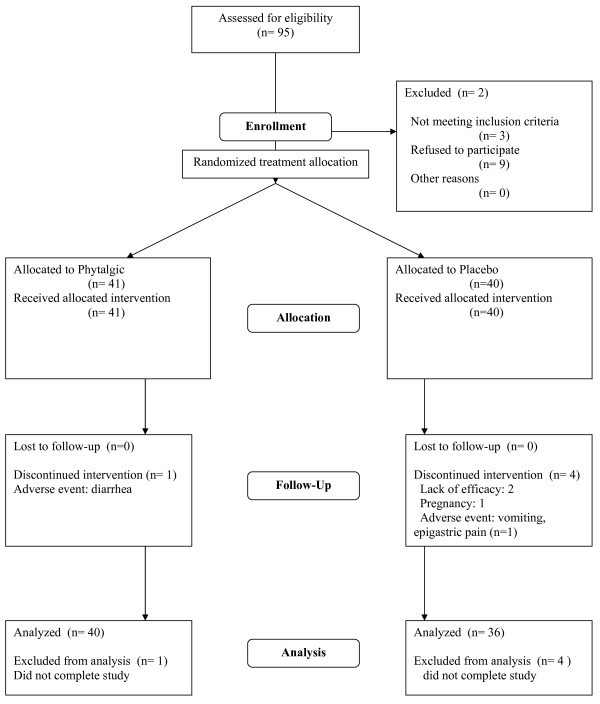
Patient disposition in the study (CONSORT diagram).

Five patients did not complete the study: one in the active treatment group because of an adverse event (diarrhoea with positive rechallenge); four in the placebo group: two for lack of treatment effect, one for adverse event (vomiting and epigastric pain), and the last became pregnant and was excluded from the study. All dropped out before the end of the first study month. None contributed any data to the study beyond inclusion. Patient disposition is shown in Figure 1.

In the active treatment group there were 14 men and 27 women, mean age was 56.8 years, (range 28 to 79). In the placebo group there were 12 men and 28 women, mean age was 57.5 (range 28 to 84). Overall 41 patients were fully assessable in the active treatment group, vs. 40 in the placebo group. The patients who dropped out of the study were not different from those who completed the study. Two patients, one in each study group, were wrongly included, their age (28) being below the inclusion limit. Both had post-traumatic knee OA, radiologically confirmed with osteophytes, combined with overweight. Removing them from the study did not change study results. One 84 year-old patient was above the age range.

There was no difference between the active and placebo groups for initial distribution of any of the studied parameters (Table 1).

**Table 1:** Patient characteristics at inclusion

	Phytalgic^®^	Placebo
Total number of subjects	41	40
Male/female	14/27	12/28
Mean Age (range), years	56.8 (28 to 79)	57.5 (28 to 84)
Osteoarthritis (n)		
One knee	13	21
Two knees	12	8
One hip	8	5
Two hips	3	4
Knees and hips	5	2
WOMAC scores at inclusion (mean ± SD)		
Pain score	215.7 ± 88.0	229.5 ± 112.1
Stiffness score	98.6 ± 50.6	97.9 ± 63.2
Function score**	688.8 ± 281.0	689.0 ± 368.9
Total score**	1000.8 ± 391.5	1014.6 ± 526.8
Ongoing treatment (n)		
Analgesics (number of users, tablets/week ± SD)	34 (19.8 ± 12.7)	35 (19.1 ± 14.4)
Single-component Paracetamol	12 (21 ± 14.7)	15 (15.4 ± 10.4)
Combined Paracetamol	19 (15.6 ± 10.9)	17 (15.1 ± 12.1)
NSAIDs: n (DDD ± SD)	23 (1.3 ± 0.9)	23 (1.1 ± 1.1)
OA Treatment*: n (DDD ± SD)	16 (1.1 ± 0.5)	12 (0.9 ± 0.4)

*OA treatment: slow-acting drugs such as diacerhein, glucosamine, chondroitin sulphate...

DDD: defined daily doses; WOMAC: Western Ontario-Macmaster University Osteoarthritis scores;

**Four patients with missing scores at baseline (two patients in each group)

Values are numbers of patients, or mean value ± SD unless otherwise indicated. There was no significant difference between the randomized study groups.

### Use of analgesics and NSAIDS

Initial use of analgesics or NSAIDs is shown in Table 1. All patients used at least one or the other: overall 69 of the 81 patients used common analgesics (mostly paracetamol alone or combined, less frequently opiates, low-dose NSAIDs or aspirin), using about 19.4 tablets equivalent to 500 mg paracetamol or 200 mg ibuprofen per week during the month of pre-inclusion; about half used NSAIDs at anti-inflammatory doses, and took slightly more than one DDD per day during the month pre-inclusion. Only 14 patients used morphine or other opioids at inclusion.

**Figure 2:**
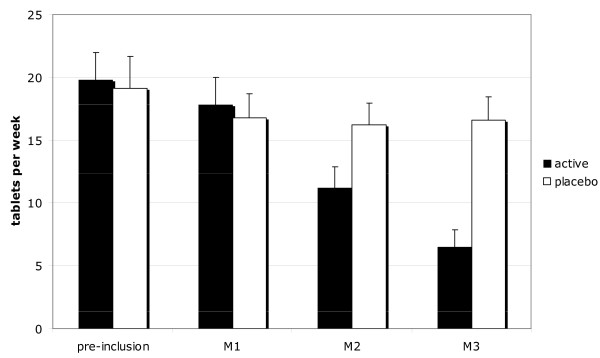
Mean use of analgesics per week in active and placebo-treated patients. (Mean number of tablets per week ± SEM). Preinclusion: the month before inclusion. M1, M2, M3, results after respectively one, two and three months of treatment.

Over the study period, the mean use of analgesics in the active arm (6.5 PET/week) vs. the placebo arm (16.5 PET/week) was significantly different (*P *< 0.001) with a group mean difference of -10.0 (95% CI: -4.9 to -15.1). It decreased significantly and regularly in patients treated with active treatment, from a mean of 19.8 tablets per week at baseline to 6.5 tablets per week at the end of the study, whereas on placebo there was only an initial decrease, from 19.1 to 16.5 tablets per week (Figure 2). The difference between the active and placebo arms was significant (*P *< 0.001) from the second month onwards.

**Figure 3:**
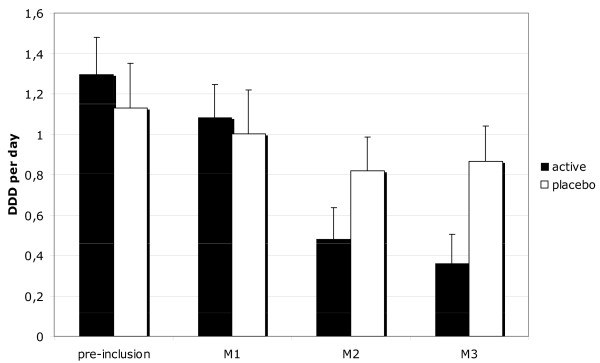
Mean use of NSAIDs in DDD per day in active and placebo-treated patients (Mean number of DDD per day ± SEM). Preinclusion: the month before inclusion. M1, M2, M3, results after respectively one, two and three months of treatment.

After a three-months follow-up, the mean use of NSAIDs in the active arm (0.36 DDD/day) vs the placebo arm (1.03 DDD/day) was significantly different (*P *= 0.02) with a group mean difference of -0.67 (95% CI: -0.16 to -1.18). Mean daily use of NSAIDs decreased in the active arm from 1.30 at baseline to 0.36 DDD/day (Figure 3) at three months. In the placebo arm it decreased from 1.13 to 1.03 DDD/day.

There was no change in the mean use of gastro-protective agents (data not shown), which concerned only eight patients in each group.

At the end of the study, the mean use of concomitant slow-acting treatment of osteoarthritis (chondroitin sulphate, diacerhein, soy and avocado insaponifiables) in the active arm (0.51 DDD/day) vs the placebo arm (0.73 DDD/day) was not significantly different (*P *= 0.82) with a group mean difference of -0.22 (95% CI: 0.13 to -0.58).

In the active arm, it decreased from 1.13 ± 0.51 DDD/day pre-treatment to 1.09 ± 0.52, 0.66 ± 0.50 and 0.51 ± 0.47 after one, two and three months of active treatment respectively (*P *respectively 0.51, 0.001 and 0.001 compared to pre-treatment). In placebo-treated patients, the values were 0.93 ± 0.44 pre-treatment, and 0.81 ± 0.51, 0.70 ± 0.45 and 0.73 ± 0.52 after one, two and three months, none of these being significantly different from pre-treatment values. Differences from baseline in the use of these drugs at the third month were significant (*P *= 0.020) between the active and placebo treatments.

### Symptoms of osteoarthritis

At the end of the study (third month), mean WOMAC scores for pain, stiffness and function in the active arm (respectively 86.5, 41.4 and 301.6) vs the placebo arm (respectively 235.3, 96.3 and 746.5) were all significantly different (*P *< 0.001) with group mean differences respectively of -148.8 (95% CI: -97.7 to -199.9), -54.9 (95% CI: -27.9 to -81.9) and -444.8 (95% CI: -269.1 to -620.4). The mean WOMAC global score in the active arm (430.1) vs the placebo arm (1085.4) was significantly different (*P *< 0.001) with a group mean difference of -655.2 (95% CI: -405.4 to -905.1).

**Figure 4:**
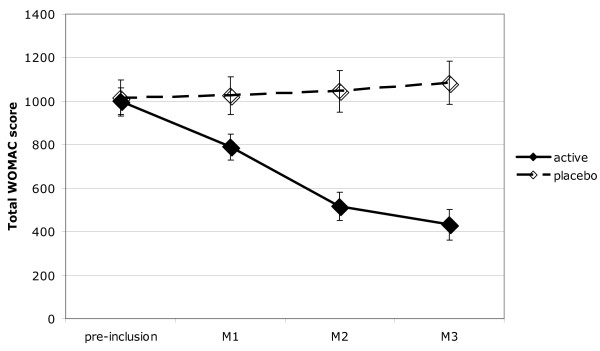
Total WOMAC scores (mean ± SEM) at preinclusion and after one, two or three months of treatment. Randomized to active or placebo treatment. Difference from preinclusion was significant at all subsequent times for active, but not for placebo.

Overall WOMAC scores did not change over the course of the study in the placebo-treated patients, whereas they decreased by more than half in active treatment patients (Figure 4). The difference between the active and placebo arms was significant from the second month on. The same pattern was found for each of the individual WOMAC score groups (Table 2).

**Table 2:** WOMAC scores at M3 in patients receiving Phytalgic^® ^and those receiving placebo

	Phytalgic^®^ n = 41	Placebo n = 40	Difference Phytalgic^® ^- Placebo	*P*
Pain				
Mean (± std)	86.5 ± 94.3	235.3 ± 136.2	-148.8 ± 26.1	<0.001
[95% CI]	[56.2; 116.9]	[194.2; 276.5]	[-97.7; -199.9]	
Stiffness				
Mean (± std)	41.4 ± 49.7	96.3 ± 71.0	-54.9 ± 13.8	<0.001
[95% CI]	[25.6; 57.2]	[74.5; 118.2]	[-27.9; -81.9]	
Function*				
Mean (± std)	301.6 ± 315.6	746.5 ± 462.8	-444.8 ± 89.6	<0.001
[95% CI]	[200.1; 403.1]	[603.1; 890.0]	[-269.1; -620.4]	
Total*				
Mean (± std)	430.1 ± 448.6	1085.4 ± 654.1	-655.2 ± 127.5	<0.001
[95% CI]	[285.0; 575.1]	[882.0; 1288.9]	[-405.4; -905.1]	

*four patients with missing scores at baseline, M1, M2 and M3 (two patients in each group).

### Tolerability and safety

Fourteen patients reported adverse events with the active treatment, one of which, diarrhoea with positive rechallenge starting on the second study day, led to withdrawal from the study. Two patients complained of eructations smelling of fish-oil, with soft stools in one. Other adverse events were pain (five patients: sciatic, sacral, lumbar, dental, scapular), infection (three patients: common cold, cystitis, lymphangitis), falls (two patients), muscle pains (one patient). All were transient and resolved with or without treatment.

Thirteen patients reported 15 adverse events during placebo treatment: these were gastroenteritis (n = 1), hypercholesterolemia (n = 1), hospitalization for systematic screening colonoscopy (two persons, both negative), dental problems (n = 3) and dental bone graft (n = 1), pregnancy (n = 1), lumbar pain (n = 1), heartburn (n = 1), cystitis (n = 1), diarrhoea (n = 1), left calf pain presumed venous (n = 1), vomiting and upper GI pain (n = 1): in this patient all analgesic and NSAID treatment and study treatment were stopped and this patient was withdrawn from the study.

## Discussion

This randomized double blind placebo-controlled clinical trial of Phytalgic^®^, a composition marketed as a food supplement, showed that after three months patients randomized to Phytalgic^® ^used fewer concomitant analgesics and NSAIDs than patients randomized to placebo, and had better WOMAC scores. This effect increased regularly over the whole study period. There were no adverse reactions attributed to the drug other than digestive (flatulence and diarrhoea, fish-smelling eructations). The prespecified endpoints were reached and the study can be seen as a first demonstration of an effect of this product, confirming anecdotal reports of patient improvement and decreased use of NSAIDs, and the validity of some recommendations [22].

Even though this is the first known trial of this specific combination, so that we really did not know what to expect, the magnitude of the effect we found, which is homogeneous over the different metrics used, was greater than we expected. The study had been powered to detect a 20% difference in the use of NSAIDs and analgesics over the study period. What we found was a greater than 50% reduction in the use of analgesics and NSAIDs.

We attempted to avoid the more common biases: Treatment allocation was random, and conformed to the usual methodology of clinical trials; the study was double-blinded, placebo and active treatments were identical in all respects. Though two patients on active treatment did complain of fish-smelling burping that might compromise blinding in these patients, removing them from the statistical analysis did not change the results of the study. Of course, this being a single-centre study should not be considered as definitive proof. Further, large scale studies will be needed to confirm these results, and possibly clarify optimal dosing and duration of treatment, maybe identify responders and non-responders, and so on.

We included run-of-the-mill patients with diverse manifestations of OA: unilateral or bilateral osteoarthritis of the knee and/or hip, which is an advantage and a drawback: an advantage because it can be seen as a *real-life *population, increasing generalisability of the results, a drawback because the patients were heterogeneous. Even though there did not appear to be any difference in response between patients with knee of hip OA, the numbers in each group were not sufficient for subgroup analysis. Any differential response between hip and knee OA would need to be explored in further studies.

The composition tested here, Phytalgic^®^, was devised empirically, from products used traditionally to treat osteoarthritis. It has been used commercially for several years, and the present study was initiated following patient reports of activity. The exact mechanism of action of the composition remains unknown, as are its possibly active components. Most of the components of Phytalgic^® ^are commonly used in practice [21,26], but clinical trials are few and often of low methodological quality. This specific combination has never to our knowledge been tested before in a randomized double-blind clinical trial, though individual components may have been: Fish oils and oils rich in omega-3 and/or omega-6 fatty acids are thought to have a possible effect on rheumatoid arthritis [27] and osteoarthritis [18], with experimental support [28]. A clinical trial of cod oil in osteoarthritis however found no clear effect [29], but the oils in the present composition come from cold-water fish rich in omega-3 and fatty acids (mackerel, herring, from the southern Pacific). Zinc is one of the essential elements for chondrocyte function, and has been associated with decreased inflammation [30] and interleukin production [31], but this would affect patients with rheumatoid arthritis more than those with OA. Nettles (*Urtica dioica*) are commonly used to treat osteoarthritis [26,32]. They may have an analgesic effect [33], and have also been found to reduce the use of NSAIDs in patients with osteoarthritis [34]. Selenium (from *Urtica dioica*) alone or associated with vitamins A, C, E has been thought to modify the symptoms or the evolution of OA [35], but recent clinical trials and meta-analyses are rather negative in this regard [36,37].

Despite these mixed results and the calls for more clinical trials such as this one [21], there seems to be an agreement that fish-oil and essential minerals are appropriate to use in patients with OA, and in this respect the preparation tested here appears to correspond with recommendations [22].

The effect of the present combination may be related to any one of the individual components, or more probably to the association of these compounds, since none of the compounds used alone seem to have demonstrated effects of the magnitude found here.

Osteoarthritis is a debilitating disease that is not usually by itself life-threatening, but is painful and crippling, and severely degrades quality of life. Its treatment is mainly symptomatic, using analgesics and non-steroidal anti-inflammatory drugs, whose adverse effects (mostly digestive or renal, but also possibly vascular) are such that they may in fact be one of the main life-threatening risks of the disease. Because of these risks inherent in the analgesic and anti-inflammatory drugs, decreasing drug use, especially of NSAIDs, could be a relevant treatment endpoint. However, reduced use of analgesics should not be associated with increased pain or reduced quality of life, but should indicate in fact less need of the drugs. This is clearly the case here: there is in our study an improvement of WOMAC scores, in all three areas covered by the scores. This symptomatic improvement was regular and increased over time, and in parallel the use of analgesics and NSAIDs decreased substantially.

WOMAC scores did not change much over the course of the study in the placebo group, though the use of analgesics and NSAIDs decreased by 20-30%. Though an improvement in OA scores with placebo is common, this is usually found in trials of single treatments, when a new treatment is given as a replacement of previous treatment or as new-onset treatment. In our patients, usual treatment was pursued. The fact that our patients were treated with analgesics and NSAIDs at baseline may also explain the relative modesty of the baseline WOMAC scores: These baseline scores are not *raw *untreated OA scores, but those obtained under usual, recognized effective treatment.

## Conclusions

This is the first randomized clinical trial of this compound. Its results need to be confirmed in other, multicentre studies, and in more varied types of OA patients, to ensure these findings are not just the result of some undetected bias, or a statistical fluke, however homogeneous and consistent they may seem.

If these results are confirmed, longer-term studies will be needed to test for the persistence of this effect. Studies in wider populations will also indicate any real gain in terms of avoided adverse reactions because of less use of analgesics and NSAIDs. Considering the size of our population and the duration of our study, we could not demonstrate this endpoint, nor did we expect to.

In the meantime, this study demonstrates that use of three capsules a day over three months of this nutraceutical compound might decrease disease scores in patients with osteoarthritis of the knee and/or hip, and reduce their use of analgesics and NSAIDs.

## Abbreviations

BOCF: baseline observation carried forward; DDD: defined daily dose; ITT: intent to treat; NSAIDs: non-steroidal anti-inflammatory drugs; OA: osteoarthritis; PET: Paracetamol 500 mg-Equivalent Tablets; SD: standard deviation; WOMAC: Western Ontario-McMaster University Osteo-Arthritis Index.

## Competing interests

The trial was funded by Laboratoires Phythea. Laurent Mallet is an employee of Laboratoire Phythea. Alain Jacquet is the main clinical investigator, and was compensated by Laboratoire Phythea for the time and activities related to the study. Nicholas Moore, Pierre Olivier Girodet and Antoine Pariente are employees of Bordeaux University and Hospitals, did not receive any compensation for the study, and have no financial or other conflict of interest with the study. Karelle Forest is a salaried employee of University of Bordeaux, and did not receive any compensation for this study.

## Authors' contributions

AJ was the main investigator in the study. He contributed to the protocol, to the data acquisition, and to data analysis. LM initiated the study within the Phythea laboratories, discussed the protocol, provided the financing for the study, and contributed to the discussion concerning putative mode of action of the product tested. NM contributed to the protocol design, to the analysis and interpretation of the study, and wrote the initial manuscript. KF did the quality control and data management for this study. AP and POG contributed to study protocol, to patient selection and to data analysis, and to the evolution of the paper. All authors read the final version of this paper and were given the opportunity to comment. All approved the final version of this paper. NM is the guarantor of this paper.

## Authors' information

AJ is an independent investigator working with the Department of Clinical Pharmacology, University of Bordeaux; AP is a university hospital practitioner in clinical pharmacology, Bordeaux University Hospitals and INSERM Clinical Investigation Centre at Bordeaux (CIC0005); POG is Master of Conferences and Hospital Practitioner in Clinical Pharmacology, University of Bordeaux, Bordeaux University Hospitals and INSERM Clinical Investigation Centre at Bordeaux (CIC0005); KF is clinical research assistant in the department of Pharmacology, University of Bordeaux, in charge of quality assurance and data monitoring; LM is Medical Director, Phythea Laboratories; NM is Professor of Clinical Pharmacology, Head of the Department of Pharmacology, and coordinator of the Clinical Investigation Centre, University of Bordeaux, Bordeaux University Hospitals, and INSERM CIC0005.
